# The Dynamism of Transposon Methylation for Plant Development and Stress Adaptation

**DOI:** 10.3390/ijms222111387

**Published:** 2021-10-21

**Authors:** Muthusamy Ramakrishnan, Lakkakula Satish, Ruslan Kalendar, Mathiyazhagan Narayanan, Sabariswaran Kandasamy, Anket Sharma, Abolghassem Emamverdian, Qiang Wei, Mingbing Zhou

**Affiliations:** 1 Co-Innovation Center for Sustainable Forestry in Southern China, Nanjing Forestry University, Nanjing 210037, China; ramky@njfu.edu.cn (M.R.); emamverdiyan@njfu.edu.cn (A.E.); 2 Bamboo Research Institute, Nanjing Forestry University, Nanjing 210037, China; 3 Department of Biotechnology Engineering, & The Jacob Blaustein Institutes for Desert Research, Ben-Gurion University of the Negev, Beer Sheva 84105, Israel; lsatish@post.bgu.ac.il; 4 Helsinki Institute of Life Science HiLIFE, Biocenter 3, Viikinkaari 1, University of Helsinki, FI-00014 Helsinki, Finland; ruslan.kalendar@helsinki.fi; 5 National Laboratory Astana, Nazarbayev University, Nur-Sultan 010000, Kazakhstan; 6 PG and Research Centre in Biotechnology, MGR College, Adhiyamaan Educational Research Institute, Hosur 635 109, Tamil Nadu, India; mathimicro@gmail.com; 7 Institute for Energy Research, Jiangsu University, Zhenjiang 212013, China; sabariswaran14@gmail.com; 8 Department of Plant Science and Landscape Architecture, University of Maryland, College Park, MD 20742, USA; anketsharma@gmail.com; 9 State Key Laboratory of Subtropical Silviculture, Zhejiang A&F University, Lin’an, Hangzhou 311300, China; 10 Zhejiang Provincial Collaborative Innovation Center for Bamboo Resources and High-Efficiency Utilization, Zhejiang A&F University, Lin’an, Hangzhou 311300, China

**Keywords:** epigenetics, transposable elements, retrotransposon, gene regulation, TE methylation, measurement of TEs, TE machine learning tool, plant stress tolerance, non-coding RNAs

## Abstract

Plant development processes are regulated by epigenetic alterations that shape nuclear structure, gene expression, and phenotypic plasticity; these alterations can provide the plant with protection from environmental stresses. During plant growth and development, these processes play a significant role in regulating gene expression to remodel chromatin structure. These epigenetic alterations are mainly regulated by transposable elements (TEs) whose abundance in plant genomes results in their interaction with genomes. Thus, TEs are the main source of epigenetic changes and form a substantial part of the plant genome. Furthermore, TEs can be activated under stress conditions, and activated elements cause mutagenic effects and substantial genetic variability. This introduces novel gene functions and structural variation in the insertion sites and primarily contributes to epigenetic modifications. Altogether, these modifications indirectly or directly provide the ability to withstand environmental stresses. In recent years, many studies have shown that TE methylation plays a major role in the evolution of the plant genome through epigenetic process that regulate gene imprinting, thereby upholding genome stability. The induced genetic rearrangements and insertions of mobile genetic elements in regions of active euchromatin contribute to genome alteration, leading to genomic stress. These TE-mediated epigenetic modifications lead to phenotypic diversity, genetic variation, and environmental stress tolerance. Thus, TE methylation is essential for plant evolution and stress adaptation, and TEs hold a relevant military position in the plant genome. High-throughput techniques have greatly advanced the understanding of TE-mediated gene expression and its associations with genome methylation and suggest that controlled mobilization of TEs could be used for crop breeding. However, development application in this area has been limited, and an integrated view of TE function and subsequent processes is lacking. In this review, we explore the enormous diversity and likely functions of the TE repertoire in adaptive evolution and discuss some recent examples of how TEs impact gene expression in plant development and stress adaptation.

## 1. Introduction

**Figure 1:**
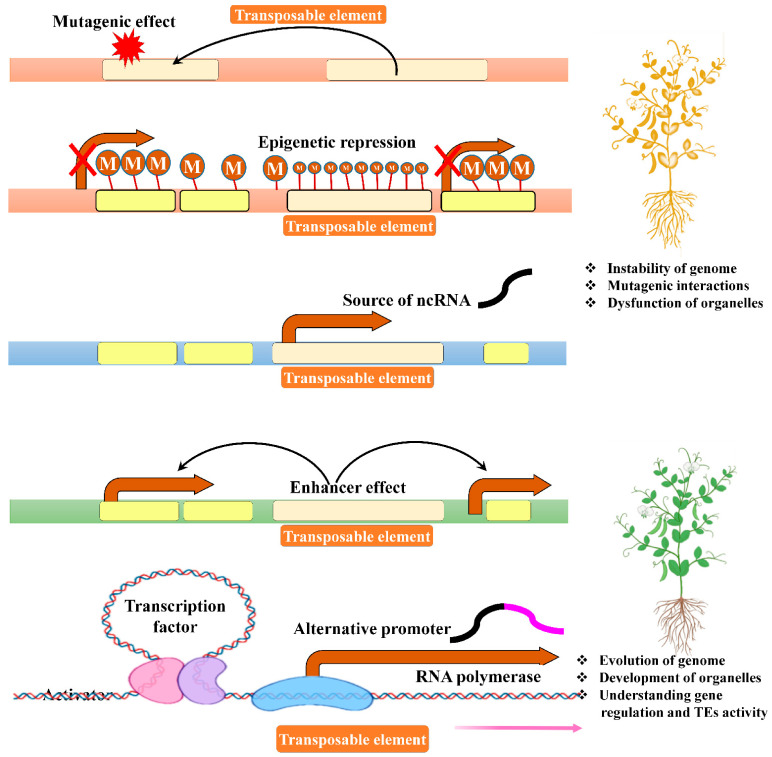
Primary regulatory roles of transposable elements (TEs). TEs are a rich source of host genome innovations. TE functions are either harmful or beneficial to the host genome, and their integration in the genome may induce deleterious mutations. Silenced TEs, mostly covered with DNA methylation, can affect the expression of nearby genes. In contrast, active TEs can act as regulatory elements by producing noncoding RNA (ncRNA) and alternative promoters. The illustration was adapted and redrawn from Jönsson et al. [43], with copyright permission from the Licensor Elsevier (Trends in Genetics: Cell Press publisher) and Copyright Clearance Center (https://www.copyright.com) (Supplementary File S1).

Transposable elements (TEs), also known as jumping genes or mobile genetic elements, are key players in plant biological systems and genome evolution [1,2,3,4,5]. TEs were previously considered as genomic parasites since these self-replicating entities are ubiquitous [6,7] and abundant in nature [8]. In recent years, several evolutionary studies in eukaryote genomes emphasized the biological significance of TEs in animals and plant genomes [9,10,11]. For example, in mammals [12] and in the model organism *Drosophila* [13], TEs have a major role in disseminating cis-regulatory elements that help the host genome regulate its own genes both in the short-term (adaptation to environmental changes) and long-term (evolutionary changes). Furthermore, TEs act as key factors in diverse genetic mechanisms, such as chromosomal changes related to recombination processes of mobile genetic elements and other elements, regulation and expression of genes, genomic evolution, and genetic instability (Figure 1) [14,15,16]. TE transpositions may even cause mutations that lead to novel functional protein-coding sequences [17,18]. For example, *Rag1* and *Rag2* are TE-derived conserved genes that catalyse V(D)J somatic recombination in the vertebrate immune system [19,20]. As a consequence of the biological significance of TEs, TEs have recently been used as an integration tool in fundamental research [21] and in gene therapy [22]. TEs, or parts thereof, can also be implemented into common molecular biology tools, such as expression vectors [23]. In addition, TEs have been suggested as new markers (together with mitochondrial polymorphisms and Y-chromosome polymorphisms) to describe the evolutionary history of a species, or even of single individuals [24,25].

However, TEs are the most erratic components in plants and are species-dependent [26,27,28,29]. The host applies several strategies to control TE activities to avoid potential deleterious actions by other TEs, such as retrotransposon elements (RTEs). While most of the long terminal repeat (LTR) RTEs were recently inserted in most plant genomes, these insertions are unique in the genome. For example, some RTEs are transcriptionally inactive under normal conditions, but under different stress conditions, most of the RTEs are active [30]. The flexible genomic alterations in RTEs can be considered suitable for most plant adaptation mechanisms under various stresses, including biotic and abiotic stress [31,32,33]. However, plants possess a potent response that restrains TE activity, leading to epigenetic silencing of these elements, which results in alteration in plant gene function [15,34,35,36]. For instance, in the African oil palm (*Elaeis guineensis*), DNA hypomethylation of a *LINE* (non-LTR RTEs), related to rice *Karma*, is linked with alternative splicing and yield loss, whereas hypermethylation near the *Karma* splice enhanced the normal fruit set [37]. Typically, TE insertion did not impact the genome or related biomolecular products because of TE silencing [38]. For instance, in *Arabidopsis* and corn (*Zea mays*), methylation of mutated TEs is not harmful to the genome [26,33,39,40]. TE silencing is caused by miRNAs or epigenetic mechanisms, such as DNA methylation or chromatin remodelling [38,41]. The addition of a methyl group to the cytosine bases of DNA to generate 5-methylcytosine is called DNA methylation [42].

Among several epigenetic mechanisms, DNA methylation and chromatin remodelling are more commonly implicated in the inactivation of TEs in plants and animals [40,44,45,46,47,48]. TEs are transcribed steadily in methylation-deficient plants and cause mutant phenotypes that are directly linked to TE insertion [14,42]. The other most significant epigenetic mechanism is chromatin remodelling. The altered chromatin structure results in constricted chromatin at the particular site of the genome where genes and transposons are inactivated, as the RNA polymerase is unable to access those sites. For example, in *Arabidopsis*, decondensed chromatin regulates the expression of small RNAs to help maintain TE methylome homeostasis during post-embryogenesis [49]. Hence, most elements are not transcribed [50]. Nevertheless, further investigations are required to understand the possible mechanisms of TEs involved in plant evolutionary processes and stress adaptation mechanisms. This review addresses TE methylation mechanisms and their significance in plant evolution and stress adaptation.

## 2. TE Classification and Copy Number in Plants

According to TE structure, the plant evolves and adapts as a consequence of dynamic changes in the TE. Based on the method of transposition (movement), TEs are classified into two major classes, class I and class II (Table 1) [51]. Class II (DNA transposons) are usually present in low copy numbers and are mobilized through a DNA intermediate by “cut-and-paste” mechanisms [52], as in the case of the *Helitrons* transposon, which is a “peel-and-paste” replicative mechanism via a circular DNA intermediate [53]. Class I transposons or RTEs are mobilized by copy-and-paste using RNA as an intermediate, whereby RNA is reverse-transcribed into cDNA then integrated into a target site of the genome [54,55,56].

Based on its structure and mechanism of integration, RTEs are further divided into different superfamilies, such as long terminal repeat (LTR) RTEs, non-LTR RTEs, and dictyostelium repetitive sequences (DIRS) [30]. LTR RTEs are the most common superfamily, contributing up to 80% of plant genome size [57], and have significantly higher copy numbers than other superfamilies and classes (Table 1).

According to Wicker et al. [51], class I (retrotransposons) do not require subclasses but superfamilies. However, class II transposons are classified into two subclasses distinguished by the number of DNA strands and do not move via an RNA intermediate. Each subclass is further classified into different superfamilies and families, with wide variations in the organization, but with shared common genetic structures and monophyletic origin. For example, the families of Ty3/*gypsy* and Ty1/*copia* are superfamilies of LTR RTEs found in virtually all major groups of eukaryotes [58]. Similarly, *Tcl*/*mariner*, hATs (hobo-Ac-Tam3), and *MULEs* (*Mutator*-like elements) are subclasses of DNA transposons that are widespread in eukaryotes [59]. Although conversion to the wild-type sequence at the insertion site can occur upon transposition, many types of transposons leave a detectable footprint upon mobilization. However, the net excision of the donor site of cut-and-paste transposons is generally challenging to detect since the donor site is converted to a normal site either by using a homolog as a template or a sister chromatid [59].

Both class I and class II TEs have autonomous (containing open reading frames, ORFs) and non-autonomous (absence of encoding potential while lacking transposition ability) TEs [12,23,60,61]. Class II autonomous TEs can encode transposase and helicase enzymes for cut-and-paste mechanisms [62]. Class I autonomous TEs can encode specialized Gag packaging proteins and reverse transcriptase for transposition [1]. The transposition-competent TEs have not only coding ability but also bear intact *cis*-acting elements that interact with the transposition complexes. LTRs (class I) and terminal-inverted repeats (TIRs) (class II) are examples of such *cis*-acting elements. Thus, autonomous elements are not dependent on any other factors for their movement [33], whereas non-autonomous TEs depend on autonomous TEs to migrate. However, non-autonomous elements can still express transposition-related proteins while lacking transposition ability [61]. For example, *Ac* (*Activator*) TEs can translocate their position as they are autonomous. In contrast, *Ds* (*Dissociation*) TEs are non-autonomous and can only be transposed by the availability of Ac or any other autonomous element [63]. The continuous transposition of TEs in the plant genome leads to significant evolutionary changes, constant divergences, and integrations that result in, as yet, uncharacterized variations in TE forms and shapes [3].

**Table 1:** Class- and family-wise examples of transposable elements (TEs) in different plant species. The table was adapted and recreated from Feschotte et al. [64], with copyright permission from the Licensor Springer Nature (Nature Reviews Genetics: Nature publisher) and Copyright Clearance Center (https://www.copyright.com) (Supplementary File S2).

Class	Subclass	Superfamily/Family	Plants	Autonomous Members	Non-Autonomous Members	Copy Number of the Entire Family	References
Class I	LTR Retrotransposons	*copia*-like	*O. sativa*	*Tos17*	-	(2–5) 30	[65]
		*copia*-like	*Hordeum* sp.	BARE-1	-	5000–22,000	[66]
		*copia*-like	*N. tabacum*	*Tto1*	-	30 (300)	[67]
		*copia*-like	*N. tabacum*	*Tnt1A*	-	>100	[68]
		*copia*-like	*Z. mays*	*Hopscotch*	-	5–8	[69]
		*copia*-like	*Z. mays*	-	*BS1*	1–5	[70]
		*copia*-like	*Z. mays*	*Opie-2*	-	100,000	[71]
		*gypsy*-like	*O. sativa*	*RIRE2*	*Dasheng*	1200	[72]
		*gypsy*-like	*Z. mays*	Magellan	-	4–8	[73]
		*gypsy*-like	*Z. mays*	*Huck-2*	-	200,000	[71]
		*gypsy*-like	*Arabidopsis*	*Athila 4*	-	22	[74]
		*gypsy*-like	*Arabidopsis*	*Ta3*	-	1	[75]
		*gypsy*-like	*Arabidopsis*	*Athila 6*	-	11	[74]
		*gypsy*-like	*Arabidopsis*	*Tar17*	-	2	[67]
	Non-LTR Retrotransposons	*LINEs; L1-clade*	*Lilium speciosum*	*Del2*	-	250,000	[76]
		*LINEs; L1-clade*	*Z. mays*	*Cin4*	-	50–100	[77]
		*LINEs; L1-clade*	*Arabidopsis*	*Tal1*	-	1–6	[78]
		*SINEs*	*N. tabacum*	*-*	*TS*	50,000	[79]
		*SINEs*	*B. napus*	*-*	*S1*	500	[80]
Class II	DNA transposons	*Mutator*	*Z. mays*	*MuDR*	*Mu1*	10–100	[81]
		*Mutator*	*Arabidopsis*	*AtMu1*	*-*	1 (4)	[82]
		*CACTA*	*Z. mays*	*Spm*	*dSpm*	50–100	[83]
		*CACTA*	*Arabidopsis*	*CAC1*	*CAC2*	(4) 20	[84]
		*hAT*	*Z. mays*	*Ac*	*Ds*	50–100	[85]
		*PIF/Harbinger*	*Z. mays*	*PIFa*	*mPIF*	6000	[86]
		*PIF/Harbinger*	Angiosperms	*PIF-like*	*Tourist*-like	Variable	[86,87]
		*Tc1/Mariner*	Angiosperms	*MLEs*	*Stowaway*-like	Variable	[88,89]

The copy numbers indicated are approximate and collected from various research articles. Those in parentheses result from transpositional activation (*Tos17* and *Tto1*) or in mutant backgrounds (*CAC* and *AtMu1*). *LINE: Long Interspersed Nuclear Element, SINE: Short Interspersed Nuclear Element*, *Ac*: *Activator*; LTR: Long Terminal Repeat, *MLE: Mariner*-*Like* Element, *Ds*: *Dissociation*, *mPIF*: *miniature P Instability Factor*, *Spm*: *Suppressor*–*Mutator*, *PIF: P Instability Factor*.

## 3. Surprising Traits of TEs

In plants, TEs are located within or near the gene or promoters. The position of the TE determines plant gene expression and other regulatory mechanisms for growth and development and stress adaptation. TEs are aligned at a suitable location in the genome through transposition [90]. The aligned position should positively interact with the organelles of the cell [91]. This location-based, genomic-level adaptation through these various shapes of TEs surprised plant biologists by their outstanding genomic parasitism, optimistic competition, and cooperation with other cellular processes [15,92]. Another significant and surprising property of TEs is the spectrum of site selection for transposition in the plant genome [12]. However, the TE selection mechanism in the genome is still unclear as TEs insertion sites are not detrimental and not strongly counter-selected [93]. This indicates that natural selection and genetics are the most significant and forceful genome-shaping factors, acting through the adequate distribution and accumulation of various TEs in the plant genome [94]. Under certain circumstances, this insertion by transposition could cause positive effects that isolate the species from native populations.

In most cases, the insertion will have little or no effect on gene activity. In some cases, such insertions might alter gene expression such that the plant is better adapted to environmental and ecological conditions. The impact of such insertions might differ significantly among species [95]. The position of some TEs in the genome is more stable than that of other TEs. This genome stability is directly related to the forces of selection [96,97,98]. Such properties of various classes of TEs have shaped the genomes of plant species, thereby maintaining genome stability and function. A clear understanding of how natural forces of selection impact the transposition of TEs in the plant genome can provide valuable insights into evolutionary processes in plant biological systems.

## 4. Contribution of TEs in the Plant Genome

The average genomic fraction occupied by TEs in plant genomes is about 50% of the entire genome. This percentage can range from 15% in small to >85% in large plant genomes (Table 2) [99,100,101]. RTEs occupy a significant portion of the plant genome and are the most significant factor in the plant genome, thus contributing to plant growth [99,102] (Table 2). This variation was reported by researchers [103,104], who examined the possible relationship between LTR-RTEs and the total physical length of the plant genome. The total genomic content of plant species is a linear function of TE content. Thus, LTR-RTEs are significant components of the plant genome and contribute to the genome differences among plants [105].

The proportion of RTEs in the total genome of several plant species is directly correlated [100,106,107]. For example, the total proportion of RTEs in the total genome of *Arabidopsis* is 14% (total genome size: 125 Mb) [108]; it is 35% in *Oryza sativa* (total genome size 389 Mb) [109], and 85% in *Zea mays* (total genome size: 2.3 Gb) [110]. Among these plant species, Z. mays contains more RTEs than any other plant species investigated thus far (Table 2) [99]. Hence, the existence of an excess volume of RTEs in *Z. mays* has gradually increased (doubled) the total genome size in the past 3 million years due to the swift propagation of RTE families [99,111]. Similarly, the genome size of *O. australiensis* has doubled due to the rapid proliferation of three LTR-RTEs families (*RIRE1*, *Kangourou*, and *Wallabi*) [112].

**Table 2:** Proportion of class I and class II transposable elements (TEs) in the total genome of different plant species [99,100,101,102,104,110,113,114,115,116,117,118,119,120,121,122,123,124,125,126,127,128,129]. The table was adapted and recreated from Ragupathy et al. [99], with copyright permission from the Licensor Elsevier (Trends in Plant Science: Cell Press publisher) and Copyright Clearance Center (https://www.copyright.com) (Supplementary File S3).

Plant Genome	Total Genome Size (Mb)	Total TE Content (% of the Genome)	Total Class I or RNA (Retroelements) (% of the Genome)	Total Class II or DNA Transposons (% of the Genome)
*Aegilops tauschii*	4.98	68.20	13.30	53.50
*Arabidopsis lyrata*	230.00	29.70	15.99	4.80
*Arabidopsis thaliana*	125.00	14.00–18.50	7.50	11.00
*Brachypodium distachyon*	355.00	28.10	23.33	4.77
*Brassica oleracea*	600.00	20.00	14.00	6.00
*Brassica rapa*	529.00	39.51	29.90	3.20
*Cajanus cajan*	833.00	51.67	19.18	4.53
*Carica papaya*	372.00	51.90	42.80	0.60
*Cicer arietinum*	738.00	49.41	45.64	9.32
*Citrus sinensis*	367.00	20.50	18.21	2.28
*Cucumis melo*	450.00	19.70	14.70	5.00
*Cucumis sativus*	367.00	24.01	12.16	1.24
*Fragaria vesca*	240.00	22.81	16.37	6.44
*Glycine max*	1115.00	58.74	42.24	16.50
*Gossypium herbaceum*	1660.00	52.10	52.00	0.10
*Gossypium raimondii*	880.00	56.95	48.99	4.54
*Gossypium raimondii*	880.00	61.30	54.90	1.50
*Hordeum vulgare*	5100.00	58.89	52.83	5.25
*Linum usitatissimum*	370.00	24.29	20.62	3.80
*Lotus japonicus*	472.00	30.80	10.4–19.23	0.97–8.10
*Malus domestica*	742.00	42.40	37.60	0.90
*Medicago truncatula*	475.00	38.00	9.60	ND
*Medicago truncatula*	550.00	30.50	26.50	3.40
*Musa acuminata*	523.00	32.63	31.17	1.42
*Oryza sativa*	389.00	34.79	19.35	12.96
*Phyllostachys edulis*	1908.00	45.45	38.20	7.25
*Populus trichocarpa*	485.00	42.00	10.30	2.50
*Populus trichocarpa*	550.00	34.90	7.02	2.10
*Pyrus bretschneideri*	527.00	53.10	45.97	12.12
*Ricinus communis*	320.00	50.33	18.16	0.91
*Secale cereale*	8090.00	69.30	64.30	5.00
*Setaria italica (Accession Zhang gu)*	510.00	46.30	31.60	9.40
*Setaria italica (Inbred Yugu1)*	510.00	40.00	25.00	ND
*Solanum lycopersicum*	900.00	63.20	62.30	0.90
*Solanum tuberosum*	844.00	62.20	32.29	3.94
*Sorghum bicolor*	730.00	62.00	54.52	7.46
*Theobroma cacao*	430.00	25.70	17.70	8.00
*Vitis vinifera*	475.00	41.40	17.04	0.43
*Zea mays*	2300.00	84.20	75.60	8.60

## 5. Distribution of TEs in the Plant Genome

Each TE is distributed in the plant genome with a specific insertion preference [130]. LTR- RTEs, such as the Ty3/*gypsy* and Ty1/*copia* superfamilies, are present in the centromere regions of the plant genome and play significant and perilous parts in the formation and function of centromeres [12,106,131]. In addition, Ty3/*gypsy* and Ty1/*copia* exhibit nested insertions, particularly in large genomes bearing a high number of elements and prefer older copies of the same family. This suggests that nesting of LTR-RTEs is not random and depends on chromatin modifications. Class II TEs can also lead to TE nesting, although nesting is common in LTR-RTEs [130].

Similarly, nonautonomous LTR-RTEs, such as *Dasheng*, are positioned in the pericentromeric regions of the genome of *O. sativa* [72]. The grapevine RTE 1 (*Gret1*) is a type of LTR retroelement. At the same time, the insertion and rearrangement of *Gret1* in *Vitis vinifera* occurred close to the region of the *VvmbyA1* gene, which led to development of colour variation in the fruit of *Vitis vinifera* [132]. Similarly, *Rider* is a type of LTR element. While *Rider* is inserted into another region, it acts as a novel regulatory element and enhances the expression of the Ruby gene, which leads to enhanced synthesis of anthocyanin production in the fruit of *Citrus sinensis* [133]. Consistently, the fruit shape of *Solanum lycopersicum* has been altered from round to oval due to the retroposition of the IQD12 gene [134]. In *Arabidopsis* (*Landsberg erecta* (*Ler*) accession early flowering), when *mutator*-like TEs are subjected to epigenetic modification, alteration in the first intron of *Flowering Locus C* (*FLC*) results in a delay in the flowering process [135]. Likewise, *Ac/Ds* are composed of autonomous and nonautonomous members of the maize hAT family, respectively. *Ac/Ds* can also stimulate structural rearrangements of other TEs in *Z. mays* [136,137,138] and can induce chromosomal rearrangements at the rice *OsRLG5* locus [139].

## 6. TE-Induced Mutations

Active TEs induce heritable mutations in the genome that have been fully characterized at both the genetic and molecular levels. Several reports also state that TEs are mutagens and may be responsible for mutation through various means, such as by inserting themselves into active genes or near genes that contain promoter and enhancer elements. Although all active genes contain at least a promoter and many are influenced by enhancers, TE insertion still causes heritable mutations or alters gene activity [1]. Therefore, TEs are considered as the most potent natural evolutionary and adaptation mediators within the genome of plant species. TEs play a critical role in adaptation and new species formation by evolution, as TE insertions generate gene (DNA) rearrangements and can act as new coding and regulatory sequences (Figure 1) [140]. The high copy number (3000 to 10,000 per genome) of both classes (I and II) of TEs have site-specific (e.g., TAA or TA) insertions or transitions in plants. Tourist and stowaway elements belonging to *MITEs* in maize and sorghum, respectively, are preferably located at the 5′ and 3′ noncoding sections in the genes of these plants [141]. Furthermore, these elements are interconnected with the regulatory portion of genes in different flowering plants [142]. In cut-and-paste transposition, a faulty repair process may seal the gap formed during transposition. Moreover, identical repetitive sequences create a problem in the pairing process, especially during meiosis [106]. In some cases, TEs may insert the stopping codon that results in the production of truncated proteins [143].

*Arabidopsis* is a genetic model plant used for evolutionary biology and mutation-related studies and has significantly contributed to our TE research. However, an in-depth analysis of the active TEs of *Arabidopsis* mutation accumulation lines showed an absence of TE-induced direct mutation [144,145]. Surprisingly, study of *Arabidopsis* mutation accumulation lines revealed the limited scale of TE-induced mutations, which were approximately 1/haploid genome/generation. TEs involved in the insertion process could be analysed through purifying selection and population genomic analyses of polymorphic TEs, which provide a partial view of TE migration or transition [93,146].

## 7. Association of RTEs with Genomes

Approximately 7.5% to 75% of the genomes of many plant species consist of RTEs (Table 2), which play a vital role in the evolutionary process. According to recent studies on genome analyses, approximately 67% of the hexaploid *Triticum aestivum* (wheat) genome is made up of RTEs, which are primarily TEs of the class I Ty3/*gypsy* and Ty1/*copia.* The chromosome content of hexaploid wheat has been improved with highly repetitive RTE elements [147]. The latest assemblage of hexaploid wheat (bread wheat) enhanced the extremely recurring RTE elements positioned in the A, B, and D sub-genomes of the species. Wheat is an important crop where repetitive RTEs occupy approximately 67% of the genome, as RTEs undergo a large amplification process [147]. Moreover, the TE proportion is very similar in the A, B, and D sub-genomes, which evolved approximately two to three million years ago (Mya) (based on molecular dating of chloroplast DNA) [148]. This two to three-million-year evolution by rapid amplification of various RTEs led to the development of an intricate, surplus, and allohexaploid genetic material. These lengthy evolutionary processes by RTEs made the genetic material of wheat by far the most prevalent and most intricate in form in the plant kingdom.

RTEs associated with plant genomes may further show both positive and negative impacts on genomic and phenotypic activities, such as alterations in gene activity and genome organization. This occurs through amending gene expression, disrupting protein-coding regions, and stimulating chromosomal rearrangements at a large scale [149]. Such RTE activities may create a mutation that expels the particular plant from its population. For example, RTEs are the predominant source of *cis*-regulatory elements and cause rapid alteration in the transcriptional unit of various genes under biotic and abiotic stresses [17,150]. Moreover, large RTEs and related repetitive elements may be involved in DNA double-strand break repair mechanisms and enhance chromosomal rearrangements through translocations, inversions, duplications, and deletions [1,17,149].

## 8. Balance between TE Expression and Repression

To ensure survival, plants and other organisms must evolve and adapt to the surrounding biotic and abiotic stresses [151]. Large portions of the genomes of many organisms are composed of RTEs that balance the expression and repression of essential gene sequences [152]. TEs are usually assumed to insert anywhere in the genome, but some TEs are biased in their insertion locations to balance both expression and repression. For example, *Athila* RTEs and other RTEs are inserted in the pericentromeric regions and less proximal regions of the chromosome arms, respectively. This suggests that these regions could help balance TE expression and repression through epigenetic modification [5,153]. Furthermore, for successful evolution, regulatory elements with TE insertions should balance gene expression, as overexpression may be a disadvantage and increased copy numbers may be unusable [154,155]. Insufficiency of enzymes encoded by TEs may explain the insufficient quantity for the transposition process. For instance, transposition of *Ppmar1* and *Ppmar2* (*Mariner*-like elements (*MLEs*) isolated from Moso bamboo) is determined by the quantity of transposases present inside the nucleus [156,157]. This suggests that *MLEs* generally have the potential to develop a self-regulatory strategy that can control their amplification and copy numbers by minimization of transposases. This is a well-known regulation mechanism known as overproduction (or overexpression) inhibition [158]. TE expression or its transposition may also be influenced by some default factors, such as chromatin, DNA alteration pathways, small interfering RNAs (siRNA), specific gene repressors under abiotic stress [5]. For example, Wang et al. [159] performed an experiment on three strains of *Arabidopsis* to demonstrate the significance of siRNAs and epigenetic processes (such as DNA methylation) to identify the balance between the expression and repression of genes. They found an optimistic correlation and interspecific alteration in gene expression of TE sequence polymorphisms and the existence of associated TEs. Small gene (<2 kb) sequences that possess conserved TEs are more stable than larger TEs inserted into adjacent gene polymorphisms. siRNAs serve to repress TEs (stopping proliferation) situated near coding genes, which leads to strong suppression of adjacent gene expression [48].

In some cases, such as the pollen of flowering plant species, the host cell could employ a cohort cell (that does not pass hereditary information to subsequent generations) produced simultaneously during the meiosis process, which ensures TE repression [160]. Moreover, the balance of expression and repression of TEs is also determined, and their degrees vary among tissue types and with the age of the organism (i.e., stage of life cycle). Furthermore, TEs are expressed only in germline cells and not in the somatic cells in many plant species. Hence, TEs are retained in the germline (also called micronucleus) and are actively deleted from the somatic macronucleus [160].

## 9. TE Transposition and Genome Stability

TEs associated with genes are transposed into other sites of the same genome with transposase enzymes and TE transposition machinery. Moreover, TEs involved in this transposition process can exist as replicates or conservative in form. In replicative transposition (copy-and-paste process), TEs are copied and relocated in the same genome, leading to duplicate TEs in the genome [1,17,59,149]. The cut-and-paste process is involved in conservative transposition, in which TEs are excised from their original position and transposed to the new position in the same genome. In this cut-and-paste process, the adjacent sequence of a neighbour gene sequence can be cut and reinserted into a new site in the same genome; this phenomenon can also be called exon shuffling. This transposition can cause damage to the genome by disrupting the expression of critical genes [161,162].

The plant can silence transposition through various mechanisms, such as via mutations in TEs, epigenetic silencing (e.g., DNA methylation), and siRNA silencing [163]. In certain situations, the transposition properties of TEs may assist the plant species to rapidly adapt to biotic and abiotic stresses and expand genome size [150,164]. For example, a heat-activated RTE in *Arabidopsis*, *ONSEN,* increases abiotic stress tolerance through a mutation in an abscisic acid (ABA) responsive gene and epigenetic mechanisms [165]. Initially in the transposition process, RTE generates its transcription by reverse transcriptase and reintegration into the genome, a process termed retrotransposition. In both cases, the transposase enzyme is involved in the insertion of TEs at another site. In retrotransposition, RTEs inhabit approximately 74% of the 240-kb maize genomes (*Adh* region). These elements comprise 11 different families from 23 members of RTEs [4,166]. In the transposition process, insertion age correlates with the retrotransposition process, as the ends of RTEs are probably identical during the element insertion mechanisms [26].

Although Barbara McClintock discovered TEs approximately 70 years ago, several studies have revealed new information about TEs in both prokaryotes and eukaryotes. It is now recognized that the excision and insertion traits of TEs can cause genetic instability in both prokaryotes and eukaryotes, which can lead to genomic innovations and facilitate the emergence of new species [167]. The effects of TEs on genetic stability remains poorly understood. Available data suggest that the genomic instability of TEs has both positive and negative impacts on the host. For example, genomic instability can increase genetic diversity, give an optimistic outcome, facilitate evolution, and involve gene regulation [18,168]. In contrast, genomic instability in plants may also lead to unusable phenotypic changes, such as flowering, yield reduction, and reduction in stress tolerance [97,169,170,171].

## 10. TE Is the Source of Non-Coding RNAs (ncRNAs)

Non-coding RNAs (ncRNAs) are a group of various RNA complexes that act as key factors in regulating gene expression. Based on the source and mode of action, ncRNAs are classified into housekeeping ncRNAs (tRNAs, rRNAs, and snoRNAs) and regulatory ncRNAs. Moreover, regulatory ncRNAs are sub-classified into small ncRNAs (siRNAs and miRNAs) and long ncRNAs (intronic ncRNAs (incRNAs) and long intergenic ncRNAs (lincRNAs)) [172]. Several theories, such as duplication, pseudogenization of protein-coding sequences, double-stranded RNAs (dsRNAs) from heterochromatin regions, evolution (genomic) from existing transposons, replication of RNA viruses, and random hairpin structures have been proposed to explain the source of different ncRNAs, especially regulatory ncRNAs [172]. However, a significant amount of ncRNAs is transcribed from TEs [173]. These ncRNAs, especially regulatory ncRNAs, can modify RNA stability, prevent RNA translation, and, most importantly, play a key role in the modulation of gene expression at transcriptional and post-transcriptional levels [172]. Interestingly, recently published literature suggests that ncRNAs may be involved in various stress responses in plants [174,175]. For instance, siRNAs are involved in transcriptional and post-transcriptional processes [176].

## 11. Role of ncRNAs in Plant Response to Abiotic Stress

TEs influence phenotype through the production of ncRNAs, which play a significant role in responding to and balancing abiotic stress. Several recent research findings have revealed that the active expression of ncRNAs, either directly or indirectly, is involved in plant responses to abiotic stress [177]. miRNA expression might be enhanced or suppressed in response to different abiotic stresses [178]. For example, salt stress in *Arabidopsis* induces miR393 expression, and miR393 is involved in repression of lateral root initiation, emergence, and elongation and increases levels of reactive oxygen species (ROS) in the lateral root [179].

Similarly, siRNAs contribute significantly to abiotic stress responses. For example, in *Arabidopsis*, nat-siRNA, along with SRO5, regulate proline metabolism through pyrroline-5-carboxylate dehydrogenase (P5CDH), which reduces the increased production of ROS under high salt stress [180]. Similarly, lncRNAs from plants exhibit a significant mimicry response to different abiotic stresses [181]. lncRNAs serve as competitive endogenous RNAs (ceRNAs) that have been overwhelmed by miRNAs. Thus, lncRNAs inhibit the interaction of the original miRNA at the target site [182]. For example, in *Arabidopsis* grown under phosphate deficiency stress conditions, lncRNA IPS1 is activated to mimic miRNA399, which inhibits binding of native miRNA399s to their target site, such as in the case of PHO2 [183]. Similarly, various types of siRNAs and lncRNAs from various plants mediate responses to various abiotic stresses (Table 3) [172].

**Table 3:** Abiotic stress response mechanisms of non-coding RNAs (siRNAs and lncRNAs) from various plant species.

Plant Species	siRNA	Mechanisms	Abiotic Stresses Induced/Suppressed	References
*Arabidopsis*	SRO5-P5CDH nat-siRNA	Regulation of proline metabolism	Salt stress ↓	[180]
*Arabidopsis*	TAS1, TAS2, TAS3 ta-siRNA	Elevated expression	Hypoxia stress ↑	[184,185,186]
*Arabidopsis*	HTT1, HTT2-TAS1	NYE	Heat stress ↑	[187,188]
*Arabidopsis*	TAS4 ta-siRNAs	Biosynthesis of anthocyanins	Phosphate deficiency ↑	[189,190]
*Arabidopsis*	TAS4-siR81(-)	Accumulation of anthocyanin	Nitrogen deficiency ↑	[190]
*Arabidopsis*	hcsiRNAs (ONSEN)	DNA methylation	Heat stress ↑	[191,192,193]
*Arabidopsis*	hcsiRNAs (HD2C, HDA6)	DNA methylation	Drought and ABA stresses ↑ and ↓	[194,195,196,197,198,199]
*Arabidopsis*	IPS1 *	miR399 target mimicry	Phosphate deficiency ↑	[183,200,201]
*Arabidopsis*	lncRNAs *	Antisense transcription	Light stress ↑	[202]
*Arabidopsis*	asHSFB2a *	Antisense transcription	Heat stress ↑	[203]
*Arabidopsis*	COOLAIR *	Chromatin remodelling	Cold stress ↑	[204]
*Arabidopsis*	lncRNAs *	Histone modification	Light stress ↑	[202]
*Arabidopsis*	COLDAIR *	Histone modification	Cold stress ↑	[205]
*Arabidopsis*	lncRNAs *	RdDM pathway	Heat stress ↑	[206]
*Arabidopsis*	lncRNAs *	RdDM pathway	Salt stress ↓	[207]
*Brassica oleracea*	nat-siRNAs	DNA methylation	Heat stress ↑	[208,209]
*Brassica rapa*	nat-siRNAs	DNA methylation	Heat stress ↑ and ↓	[209]
*Brassica rapa*	lincRNAs *	miRNA precursors	Cold and heat stresses ↑ and ↓	[210]
*Craterostigma plantagineum*	CDT1-siRNA	NYE	Dehydration stress ↑	[211]
*Manihot esculenta*	2 nat-siRNA, 3 ta-siRNAs	NYE	Cold stress ↑ and ↓	[212]
*Oryza sativa*	lncRNAs *	target mimicry	Phosphate deficiency ↑ and ↓	[213]
*Phaeodactylum tricornutum*	pti-MIR5472 *	miR5472 precursors	Phosphate deficiency ↑	[214]
*Phaeodactylum tricornutum*	pti-MIR5471 *	miR5471 precursors	Phosphate deficiency ↑	[214]
*Populus tomentosa*	lincRNAs *	miRNA precursors	Nitrogen deficiency ↑ and ↓	[215]
*Populus tomentosa*	lincRNAs *	Antisense transcription	Nitrogen deficiency ↑ and ↓	[215]
*Populus trichocarpa*	lincRNA1128 *	ptc-miR482a.1 target mimicry	Drought stress ↓	[216]
*Populus trichocarpa*	lincRNA1393 *	ptc-miR6459b target mimicry	Drought stress ↓	[216]
*Populus trichocarpa*	lincRNA3018 *	ptc-miR399i target mimicry	Drought stress ↓	[216]
*Populus trichocarpa*	lincRNA2752 *	ptc-miR169o target mimicry	Drought stress ↑	[216]
*Populus trichocarpa*	lincRNA1795 *	ptc-miR476a target mimicry	Drought stress ↓	[216]
*Populus trichocarpa*	lincRNA20 *	ptc-miR476a target mimicry	Drought stress ↑	[216]
*Populus trichocarpa*	lincRNA2623 *	ptc-miR156k target mimicry	Drought stress ↓	[216]
*Populus trichocarpa*	lincRNA2623 *	ptc-miR156c target mimicry	Drought stress ↓	[216]
*Populus trichocarpa*	lincRNA967 *	ptc-miR6462e target mimicry	No response to drought stress	[216]
*Populus trichocarpa*	lincRNA2762 *	ptc-miR156k target mimicry	Drought stress ↓	[216]
*Populus trichocarpa*	lincRNA1449 *	ptc-miR156k target mimicry	No response to drought stress	[216]
*Populus trichocarpa*	lincRNA179 *	ptc-miR156a target mimicry	No response to drought stress	[216]
*Populus trichocarpa*	lincRNA2198 *, lincRNA2131 *, lincRNA2085 *, lincRNA2962 * lincRNA1534 *, lincRNA1039 * lincRNA2962 *	NYE	Drought stress ↑	[216]
*Solanum lycopersicum*	lncRNAs *	RdDM pathway	Salt and drought stresses ↓	[217]
*Triticum aestivum*	002061_0636_3054.1 siRNA	NYE	Heat, NaCl, and dehydration ↓	[218]
*Triticum aestivum*	005047_0654_1904.1 siRNA	NYE	Heat, NaCl, and dehydration ↓	[218]
*Triticum aestivum*	005047_0654_1904.1 siRNA	NYE	Cold stress ↑	[218]
*Triticum aestivum*	080621_1340_ 0098.1 siRNA	NYE	Cold stress ↑ and heat stress ↓	[218]
*Triticum aestivum*	007927_0100_2975.1 siRNA	NYE	Cold, NaCl, and dehydration ↓	[218]
*Triticum aestivum*	ta-siRNA TAS3a-5^0^D6 (+)	Auxin signalling pathway	Cold stress ↑	[219]
*Triticum aestivum*	TalnRNA5 *	ta-miR2004 precursors	Heat stress ↑	[218,220]
*Triticum aestivum*	TahlnRNA27 *	ta-miR2010 precursors	Heat stress ↑	[218,220]
*Triticum aestivum*	TalnRNA21 *, TahlnRNA3 *, TahlnRNA14 *, TahlnRNA19 * TahlnRNA36 *, TahlnRNA41 * TahlnRNA42 *, TahlnRNA47 * TahlnRNA52 *	siRNA precursors	Heat stress ↑	[218,220]
*Zea mays*	lncRNAs *	siRNA precursors and antisense transcription	Drought stress ↑	[221]

Star symbol “*” indicates lncRNA; no symbol indicates siRNA. Up arrow “↑” indicates that siRANs/lncRNAs are enhanced in response to the corresponding abiotic stress while the down arrow “↓” indicates that siRANs/lncRNAs are suppressed in response to the corresponding abiotic stress. NYE indicates that the mechanism/process of that particular siRNA or lncRNA has not yet been established. RdDM, small RNA-directed DNA methylation.

## 12. Epigenetic Effects of TEs

**Figure 2:**
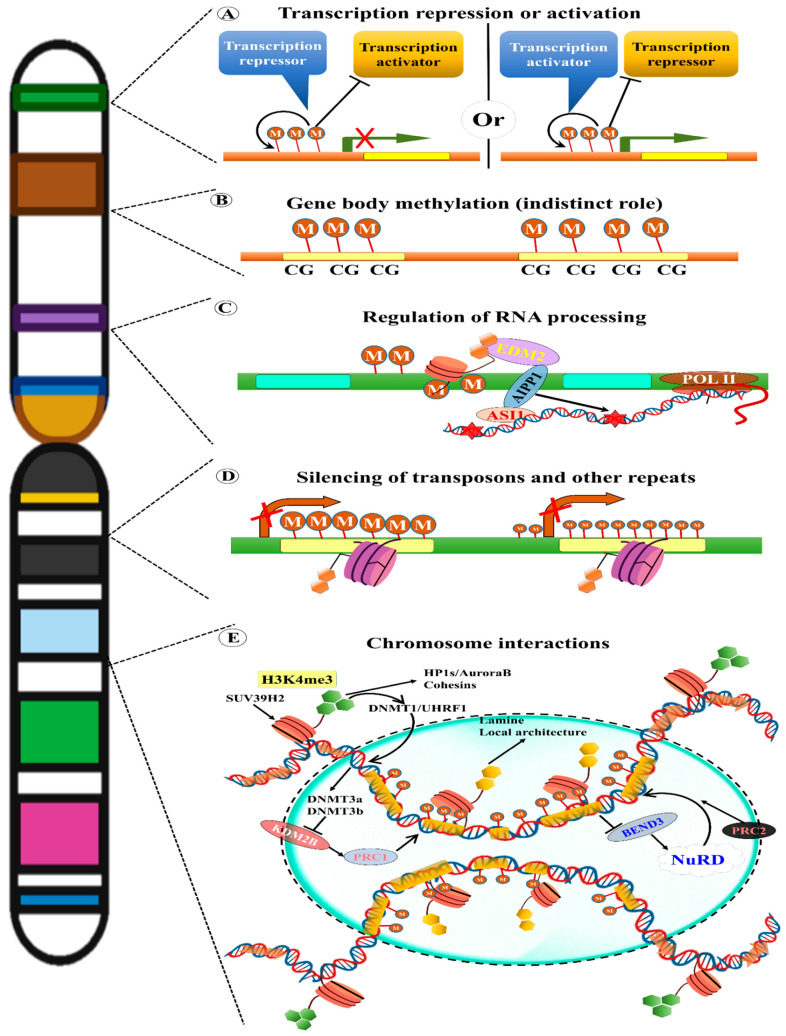
Cellular functions of DNA methylation (m) in the plant genome. DNA methylation regulates transposon activation, gene regulation, and chromosome interactions. (**A**) Methylation in the gene promoter either represses or activates transcription [229,230,231,232,233]. (**B**) Gene body methylations mainly occur in the CG context, although its function remains unknown [42,231,234,235,236]. (**C**) DNA methylation in heterochromatin regions causes the ASI1-AIPP1-EDM2 complex to enhance polyadenylation sites (red stars). ASI1 binds RNA and associates with chromatin, and EDM2 catches demethylated histone H3 lysine in the heterochromatin region [159,237,238,239]. (**D**) The methylation of transposons and other DNA repeats mainly occurs in pericentromeric heterochromatin regions [231,235]. (**E**) Chromosome interactions among pericentromeric and heterochromatin islands are regulated by DNA methylation, and repressive chromatin regions are characterized by abundant transposons and small RNAs [240,241]. ASI1, anti-silencing 1; AIPP1, immunoprecipitated protein 1; EDM2, enhanced downy mildew 2; POL II, RNA polymerase II. The illustration was adapted and redrawn from Zhang et al. [42], with copyright permission from the Licensor Springer Nature (Nature Reviews Molecular Cell Biology: Nature publisher) and Copyright Clearance Center (https://www.copyright.com) (Supplementary File S4).

**Figure 3:**
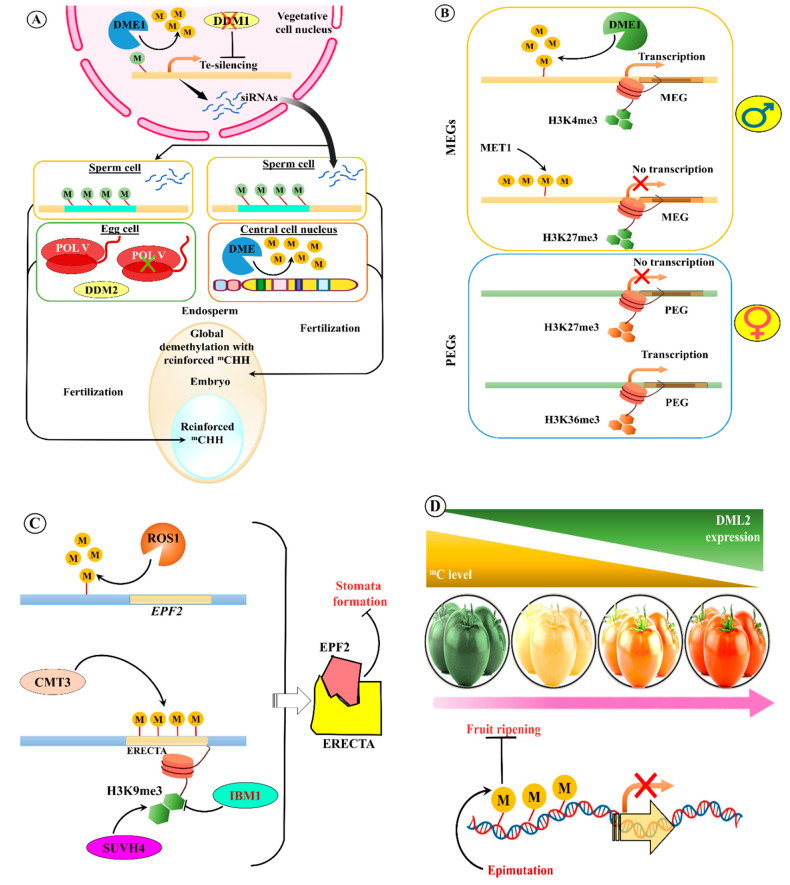
Functions of transposable element (TE) methylation in plant growth, stomata formation, and fruit ripening. (**A**) In the vegetative cell (male gamete) of *Arabidopsis*, the TE is silenced by DME-mediated DNA methylation by downregulating the chromatin remodeller DDM1. Small interfering RNAs (siRNAs) derived from TE transcripts travel from the vegetative cell to the sperm cells to reinforce global demethylation (m) in the endosperm with reinforced CHH methylation (H represents A, T, or C) [160,242,243,244,245]. (**B**) Gene imprinting in the endosperm occurs either at MEGs or PEGs through DNA and histone H3 lysine methylations [246,247,248]. (**C**) Methylation at the promoter of the gene encoding epidermal patterning factor 2 (EPF2) that suppresses stomata formation is pruned by ROS1, whose mutation silences the EPF2 or the ERECTA genes, thus resulting in stomata formation in *Arabidopsis* [249,250]. (**D**) Gradual expression of DML2 during tomato fruit ripening reduces 5-methylcytosine (mC) DNA methylation at several genes (such as CNR, involved in fruit ripening) and epimutation of those genes inhibits fruit ripening [42,229,251]. DME, transcriptional activator demeter; DDM1, decreased DNA methylation 1; MEGs, maternally expressed genes; PEGs, paternally expressed genes; ROS1, repressor of silencing 1; DML2, DNA demethylase DME-LIKE 2; MET1, methyltransferase 1. The illustration was adapted and redrawn from Zhang et al. [42], with copyright permission from the Licensor Springer Nature (Nature Reviews Molecular Cell Biology: Nature publisher) and Copyright Clearance Center (https://www.copyright.com) (Supplementary File S4).

As previously mentioned, all types of TEs from both classes have a unique role in genome instability and evolution and organism adaptation to abnormal conditions [222]. Nevertheless, insertion or transposition of TEs in normal conditions may cause harmful effects to organisms, including plants. Hence, under normal conditions (i.e., absence of mutations or biotic or abiotic stress), TEs are silenced or inactivated by epigenetic silencing mechanisms, such as DNA methylation or suppressive chromatin alterations (Figure 2) [223]. The epigenetic silencing process is more active in plants than in any other organisms. In this process, TEs can be in an inactive form, when the epigenetic silencing process is turned off, or in alleviated conditions, such as under mutant backgrounds and biotic or abiotic stress [104,224]. Recently, several studies have revealed that the promoter sequence of TEs enhances expression of genes situated nearby in plants and how this expression is controlled by epigenetic regulation, which mediates phenotypic diversity and adaptation (Figure 3) [150,225].

In some eukaryotic organisms, epigenetic effects can also participate in the proliferation and accumulation of TEs, leading to an enlargement in genome size, in which siRNA-mediated pathways can occur and end with DNA methylation in TEs [1].

In eukaryotes, biochemical modifications of DNA that lead to chromatin remodelling via histone binding are known as epigenetic modifications. These modifications provide information on gene regulation. In general, histone lysine and arginine residues are subjected to epigenetic modification. Several types of lysine residues (H3K4, H3K9, and H3K27 with mono/di/tri-methylation) have been extensively studied in animals and plants. Among these types, H3K9me2 is associated with TE methylation [33]. These suppressive epigenetic effects promote packaging of chromatin into compacted nuclear partitions of the cell [226]. In eukaryotes, especially in plants, the epigenetic silencing mechanisms directly act on TEs via the small RNA-directed DNA methylation (RdDM) pathway. Briefly, the siRNA matching regions of TEs are targeted by either AGO4 or AGO6 directed by siRNA. These targeted regions (scaffolding RNA) are transcribed by polymerase V [227,228]. These scaffolded dsRNA elements react with methyltransferases DRM1 and DRM2, leading to the methylation of TEs [226].

## 13. TE Methylation

**Figure 4:**
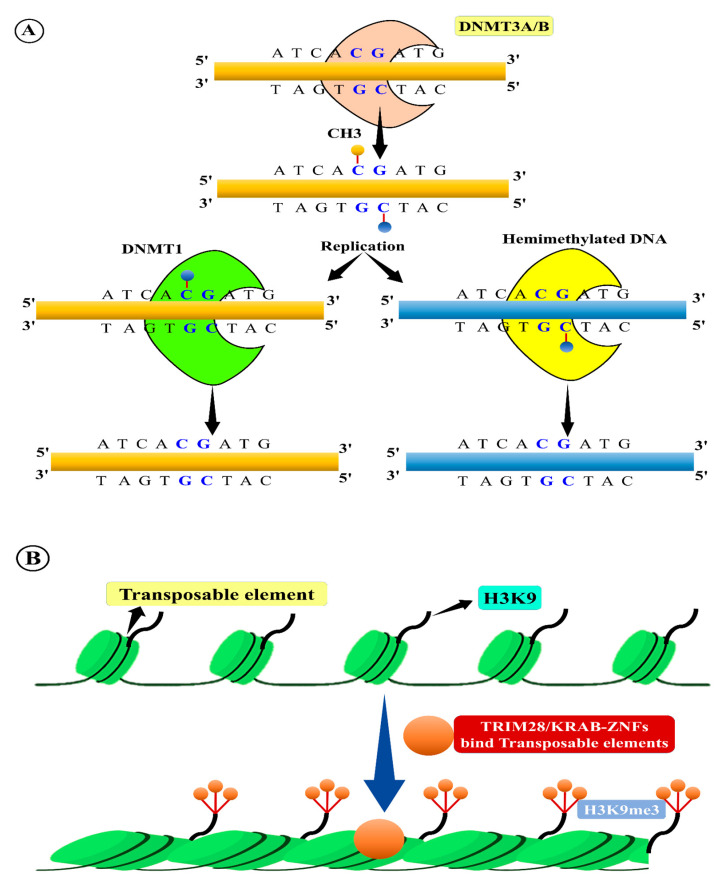
Transposable elements (TEs) are suppressed by DNA and histone methylations. (**A**) TE methylation is most commonly found in the CG context. The de novo DNA methylation is performed by DNA methyltransferases DNMT3A and 3B; the pattern of DNA methylation is maintained by DNMT1 by adding a methyl group to the newly synthesized DNA strand (a complementary strand of the hemi-methylated DNA strand), thus ensuring that the epigenetic modifications are inherited by the daughter cell. (**B**) Nucleosomes are made up of DNA and eight histone proteins. These proteins can be modified in several ways for chromatin accessibility, thereby either activating or inactivating gene expression (gene imprinting). TRIM28, a silencing complex, recognizes KRAB-ZNFs (Kruppel-associated box zinc-finger proteins), which contain a TE-binding domain and deposits H3K9me3 on TE (euchromatin region), thus causing TE repression and heterochromatin formation. The illustration was adapted and redrawn from Jönsson et al. [43], with copyright permission from the Licensor Elsevier (Trends in Genetics: Cell Press publisher) and Copyright Clearance Center (https://www.copyright.com) (Supplementary File S1).

As TEs possess self-replication potential and exist as genomic parasites, they can cause detrimental effects on essential active genes and generate ectopic recombination of DNA. These damaging effects can be avoided and controlled by epigenetic silencing, such as through DNA methylation [193]. siRNAs are interconnected with various TEs and act as mediators and stimulate DNA methylation [252]. This DNA methylation may lead to suppression of transposition through transcription reduction, along with the formation of loops among DNA and histone methylations (Figure 4) through siRNAs [253]. For example, siRNA-mediated epigenetic modification of TEs results in a delay of the flowering process in *Arabidopsis* [135]. This suggests that TE epigenetic modification regulates *FLC* expression. Hence, these siRNAs act as a strong substitute for DNA methylation in TEs, and siRNA-targeted TEs have strong effects on nearby gene transcription than those without. In some plant species, the cytosine methylation process occurs at CG, CHG, and CHH (H represents A, T, or C) sites of TEs. Most of these sites are unmethylated, and some sites (approximately 15%) are similar to DNA methylation patterns. Interestingly, siRNA-mediated DNA methylation can spread about 500 bp into unmethylated neighbouring TEs. In the case of DNA methylation in euchromatin TEs, it can spread approximately 200 bp beyond the siRNA target positions. This depends on the effect of siRNAs on the expression of proximal genes that are 400 bp in size [104,254,255].

In most cases, siRNA-mediated methylated TEs are probably situated fewer base pairs away from active genes than the location of unmethylated or partially methylated TEs. A possible reason for the partial methylation of TEs is the nucleotide composition of siRNAs. This phenomenon suggests that under unfavourable conditions, such as biotic or abiotic stress, active TEs are involved in the evolutionary process. In normal circumstances, TEs have been targeted by siRNAs for DNA methylation of cytosine to maintain genomic stability of the plant under usual conditions [42]. Moreover, to maintain TE methylome homeostasis in *Arabidopsis*, altered chromatin structure also increases siRNA production from heterochromatic TEs during post-embryogenesis [49].

## 14. TE Methylation in Plant Evolution

Since DNA methylation is positively correlated with repetitive sequences, such as RTEs and centromeric repeats non-randomly distributed across the entire plant genome, it is also enriched in centromeres in replicated regions [256]. Active TEs are mutagenic and disrupt genes, regulatory regions, and genome integrity. In contrast, the remaining new RTEs are silent and permanently or partially disabled [257]. One of the earliest known functions of the DNA methylation pathway is the inhibition of RTEs (Figure 3). In plant genomes, RTEs have significantly higher DNA methylation levels than non-coding regions (specifically CHG and CG) across all contexts [258], but some RTEs can easily escape host silencing by activating anti-silencing factors [30]. Maintenance of LTR-RTE silencing in *Arabidopsis* is based on a combination of RdDM and RNA-independent mechanisms. TE silencing accepts a distinct chromatin state. For instance, silent or increased histone H3K9 and DNA methylation in conjunction with H3 lysine results in TE suppression in *Arabidopsis*, thus protecting the genomes from TE transposition and genome instability [259]. This distinctive three-layered state of silent heterochromatin is distinguishable from the polycomb gene cluster transcribed and active heterochromatin gene expression and is linked to the *Arabidopsis* genome [260]. There can be several different reasons for the collapse of structures and reactivation of previously silenced TEs [261].

Changes in environmental conditions may lead to RTE reactivation. Alternatively, polyploidy and hybridization may cause another kind of systematic shock for RTE activation [262]. Polyploidy frequently occurs in plant genomes, making the periodic expansion of RTEs possible. For example, autopolyploidy promotes retention of TEs instead of eliminating them. Eukaryotic species seem to be linked to large population sizes, and small genomes are unusual for the few organisms known to have lost cytosine methylation. Active transpositions of DNA methylation may be less effectively eliminated in such populations [263]. Moreover, DNA methylation and gene expression patterns must be understood to understand gene expression. Although DNA methylation patterns are conserved across organisms, promoter DNA methylation is widely divergent. DNA methylation in genes and promoters are perhaps the most well-known DNA methylation pattern in plants [264].

## 15. TE Methylation in Plant Stress Response

Epigenetic modifications, including DNA and histone methylation, play a significant role in managing stress responses in plants through memory of abiotic and biotic stress factors. DNA methylation is a primary mediator of plant stress responses.

### 15.1. Abiotic Stress

**Figure 5:**
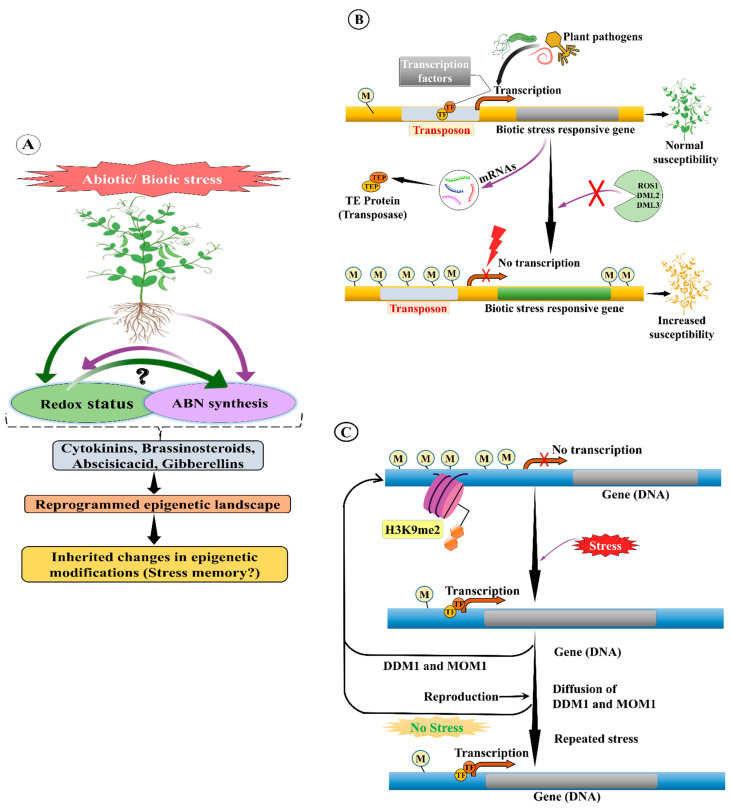
Epigenetic modifications under stress conditions and possible stress memory. (**A**) Both biotic and abiotic stresses can induce or change DNA methylation (5-methylcytosine, mC) and induce other epigenetic changes in the genome; such modifications are associated with the expression of stress-response genes, which conversely may lead to epigenetic processes. Reprogrammed epigenetic modifications (stress memory) are inherited by the offspring. (**B**) In *Arabidopsis*, ROS1, DML2, and DML3 remove DNA methylation, thus collectively regulating stress responsive genes in their vicinity. Defects in demethylases, such as ROS1, DML2 and DML3, exhibit increased susceptibility to the fungal pathogen *Fusarium oxysporum* [315]. (**C**) During *Arabidopsis* recovery from heat stress, DDM1 and MOM1 regulate the deletion of stress-induced epigenetic memory. Mutations in DDM1, a chromatin remodeller, assuages transcriptional silence with a significant loss of DNA methylation. MOM1 intermediates facilitate transcriptional silence via an unknown mechanism without loss of DNA methylation. Dysfunction of DDM1 and MOM1 in heat stress-induced gene de-silencing can be inherited in plants exposed to repeated stress [316]. ROS1, repressor of silencing 1; DMEL2 and DML3, transcriptional activator demeter (DME)-Like 2 and 3, respectively; DDM1, decreased DNA methylation 1; MOM1, morpheus molecule 1; H3K9me2, demethylated histone H3 lysine 9. The illustration was adapted and redrawn from Zhang et al. [42], with copyright permission from the Licensor Springer Nature (Nature Reviews Molecular Cell Biology: Nature publisher) and Copyright Clearance Center (https://www.copyright.com) (Supplementary File S4).

Under both abiotic stress (such as extremes of temperature, salinity, low nutrient levels) and under normal conditions, recent studies have shown variable expression of epigenetic gene regulators depending on the local environment, thus demonstrating the need for epigenetic regulation (Table 4) [265]. Epigenomic reprogramming research on histone-associated chromatin and DNA modification has shown that plants exhibit a genome-wide reorganization response to stress [266]. A recent study on drought response in *Arabidopsis* revealed that trimethylation at lysine 4 on histone H3 (H3K4me3/H3K9me2) is complex and directly correlates with gene expression in stressed cells (Figure 5C) [267]. Increasing histone H3 phosphorylation at alkaline pH also helps maintain heterochromatin structure. H3 threonine 3 (H3T3ph) also tends to interact with H3K4me3 during osmotic stress [268], and this could potentially impact gene expression; this has previously been proposed for histone deacetylase HDA9. The epigenomic environment also contains the repressive H3K27me3 as a partial result of priming in *Arabidopsis* [269]. DNA methylation requires a specific histone H1 variant, and two DEAD-box helicases are needed for the epigenetic silencing of gene expression in plants, leading to stress [270]. *Arabidopsis* mutants defective in all stages of the RdDM pathway or CHG maintenance have an altered stomatal index or aversion to moisture starvation [271]. This supports the hypothesis that DNA methylation regulates abiotic gene expression. Drought in several plant species leads to substantial remodelling of DNA methylation, which allows plants to respond more effectively to recurring stress and prepares offspring for future stress responses [272]. However, in this case, modifying DNA methylation still seems to be essential to regulate neighbouring gene expression [273]. Phosphate starvation induced high-level TE methylation in rice but had a very limited effect in *Arabidopsis*, suggesting species-specific TE methylation in response to stress [274].

**Table 4:** Various roles of DNA methylation in plant responses to abiotic stresses [275].

Abiotic Stress	Plants	Changes in DNA Methylation Levels	Major Effects	References
Cold stress	*Arabidopsis*	Enhanced methylation in the ALN promoter	Promotes seed dormancy	[276]
Cold stress	*Arabidopsis*	Variation in ICE1 methylation	Cold tolerance divergence in different accessions	[277,278]
Cold stress	*B. rapa*	Decreased DNA methylation levels in the *BramMDH1* promoter	Increased heat tolerance and growth rate	[279]
Cold stress	*B. rapa*	Demethylation of *BrCKA2* and *BrCKB4*	Regulation of floral transition. Regulation of temperature-dependent sex determination	[280]
Cold stress	*Cucumis sativus*	Demethylation of CHH sites	Regulation of temperature-dependent sex determination	[281]
Cold stress	*Rosa hybrida*	Enhanced CHH methylation of the RhAG promoter	Regulation of floral organ development	[282]
Drought stress	*Arabidopsis*	Increased 5mC methylation partly depending on H1.3	Adaptive response to water deficiency	[283]
Drought stress	*Brachypodium distachyon*	Decreased global 5mC while *Bacillus subtilis* strain B26 inoculation increases	Increased drought stress resilience	[284]
Drought stress	*G. hirsutum*	Global hypermethylation in all three contexts	Acclimation to drought stress	[285]
Drought stress	*O. sativa*	Differential 5mC methylation alterations	Constitutive drought tolerance	[286]
Drought stress	*Populus trichocarpa*	Increased methylation of upstream and downstream 2 kb and TEs	Regulation of drought responses	[287]
Drought stress	*Z. mays*	Suppression of *ZmNAC111* by MITE through RdDM	Natural variation in maize drought tolerance	[288]
Heat stress	*Arabidopsis*	Altered methylation of transposon remnants	Regulation of basal thermotolerance	[206]
Heat stress	*Arabidopsis*	Changes in genome-wide CHH-methylation patterns	Natural adaptation to different temperatures	[289]
Heat stress	*B. napus*	DNA hypomethylation	Regulation of heat stress responses in cultured microspores	[290]
Heat stress	*Brassica napus*	Increased DNA methylation in heat-sensitive genotypes	Adaptation to heat stress	[291]
Heat stress	*Glycine max*	Hypomethylation in all contexts	Affects the expression of genes or TEs under heat stress	[292]
Heat stress	*Gossypium hirsutum*	Reduced DNA methylation level in a heat-sensitive line	Microspore sterility	[293,294]
Heat stress	*O. sativa*	Decreased DNA methylation levels of *OsFIE1*	Regulation of seed size under heat stress	[295]
Heat, salt, cold stresses	*O. sativa*	Increased 6mA levels in heat and salt stress, decreased 6mA levels in cold stress	Regulation of plant responses to environmental stresses	[296]
Salt and drought stresses	*S. melongena*	Expression changes of C5-MTases and demethylases	Response to salt and drought stresses	[297]
Salt and drought stresses	*Solanum lycopersicum*	Activation of Rider retrotransposon	Modulation of salt and drought stress responses	[298]
Salt stress	*B. napus*	Decreased methylation in the salinity-tolerant cultivar but increased methylation in the salinity-sensitive cultivar	Acclimation to salt stress	[299]
Salt stress	*O. sativa*	Decreased 5mC levels in the *OsMYB91*promoter	Enhanced salt tolerance	[207]
Salt stress	*O. sativa*	Increased methylation level of the *osa-miR393a* promoter	Improved salt tolerance	[300]
Salt stress	*T. aestivum*	Increased 5mC levels in *TaHKT2;1* and *TaHKT2;3*	Improved salt tolerance	[301]
Salt stress	*Triticum aestivum*	Reduced methylation levels in the promoter of salinity-responsive genes	Contributes to superior salinity tolerance	[302]
Salt stress	*Zea mays*	Increased methylation of root *ZmPP2C* and demethylation of leaf *ZmGST*	Acclimation to salt stress	[303]
Salt, heat and drought stresses	*O. sativa*	Activation of an LTR retrotransposon, HUO	Modulation of stress responses	[304]

### 15.2. Biotic Stress

When compared with abiotic stress, less information is available on DNA methylation and histone post-translational modifications in response to biotic stress. Recent literature indicates that both necrotrophic and biotrophic pathogens are involved in changes to chromatin structure [305]. Chromatin modification is another layer of regulation for plant disease resistance. E3 ubiquitin ligase genes and histone monoubiquitination 1 (*HUB1*) and *HUB2* regulate the expression of R genes, which induce constitutive immune responses in an *Arabidopsis* mutant. Histone ubiquitination is directly induced at the R gene locus [306]. Loss of histone deacetylase HDA19 mediates *Arabidopsis* immune responses to the pathogen *Pseudomonas syringae* pathovar tomato (Pst) strain DC3000 [307]. Silent or suppressed genes in stress regulation are characterized by the dimethylation and trimethylation of histone H3 Lys 27 (H3K27me2/3).

The rice gene, *Jumonji C* (*jmjC*) histone lysine protein gene (JMJ705) encoding histone lysine demethylase is involved in reversing Lys DNA methylation. In transgenic plants, increased JMJ705 expression removes H3K27me3 from defence-related genes, induces their expression with the aid of jasmonic acid, and improves resistance to the bacterial blight disease pathogen *Xanthomonas oryzae* pathovar oryzae [308]. In contrast, impaired JMJ703 activity raised levels of H3K4me3 and reactivated two families of non-LTR-RTE, and loss of JMJ703 did not change silencing of TE silencing [309]. This suggests that histone modifications are involved in TE silencing to regulate the plant immune response. It is also fascinating to note that the role of TEs is also important in plant pathogens to facilitate infection. For example, the ascomycete fungal pathogen *Leptosphaeria maculans* secretes an arsenal of small, secreted proteins (SSPs) that act as effectors to modulate host immunity to facilitate infection in *B. napus*. Chromatin-based transcriptional regulation of SSP-encoding genes associated with TEs in fungi impacts disease development during infection [310].

Many differentially methylated stress-response genes were discovered in plants exposed to different pathogens. Differentially methylated regions in the genome are also linked to gene expression. Mutations in the non-CG methyltransferases (DRM1, DMR2, and CMT3) and the CG methyltransferase (MET1) lead to genome-wide hypomethylation and pleiotropic developmental defects [311]. However, the *met1* and the *drm1*, *drm2*, and *cmt3* (*ddc*) mutants showed more disease resistance to the bacterial pathogen *P. syringae* pv. tomato DC3000 (Pst). These dynamic changes in DNA methylation and the functional consequences of differential methylation in regulating defence-related genes following pathogen attack in *Arabidopsis* are facilitated by TEs. In the *Arabidopsis* triple mutant *rdd* (*ros1 dml2 dml3*), defence-related genes are typically downregulated and therefore susceptibility to the fungal pathogen *Fusarium oxysporum* is increased. These genes in the mutant contain hypermethylated TE in their promoters. In contrast, these promotors are actively demethylated in the wild-type strain. Furthermore, *ROS1*, *DML2*, and *DML3* demethylase activities are linked to fungal disease resistance, and DNA demethylation of TE sequences is largely regulated by *ROS1* [312]. In addition, DNA methylation can prime TEs to cause activation of epigenetic transducers and can also directly induce gene silencing. Repeat components of DNA regions are known as DNA methylation interferes with expression of some biotic stress response genes. Loss of TE methylation also makes it easier to start the transcription process [313]. DNA methylation regulates stress-related genes by selective suppression of active TEs in their regulatory regions [314]. However, in addition to these mechanisms, a full understanding of epigenetic changes is also essential to better understand new key factors underlying plant stress responses. For example, TE methylation changes may lead to the activation of the SA signalling pathway to trigger widespread cell death during biotic stress. However, no direct evidence linking cell death to differential methylation has been observed.

## 16. Detection of TE Modifications and Measurement of TE Expression

Detecting TE modifications and measuring TE expression can facilitate understanding how TEs alter gene expression. A wide range of molecular techniques and analytical approaches are available to assess TE expression and modifications. However, these approaches should be carefully considered before implementation [317]. Analysis of TE sequencing results or TE-derived reads is challenging, as TEs are usually present in multiple copies in the plant genome, and ncRNAs and several mRNA genes are derived from TEs. However, there are several methods to detect TE methylation. These include methylation-sensitive amplified polymorphisms (MSAPs), methylation-specific PCR (MSP), sequencing of specific genes, and high-performance liquid chromatography (HPLC). However, these techniques are not suitable for broad identification of TE-modified sites. Whole genome bisulfite sequencing (WGBS) and reduced representation bisulfite sequencing (RRBS) are widely used methods to study TE modifications. Standard methods used for next-generation sequencing (NGS) are becoming routine. Several low-cost NGS platforms, including 454 sequencings, Illumina Genome analyser, Illumina, HeliScope Single Molecular Sequencer, Helicos BioSciences, and Nanopore sequencing are available to systematically study TE methylation [318]. Similar to DNA, RNA also undergoes various modifications (known as epitranscriptomics) and plays a significant role in biological processes [319]. This will lead to new discoveries in TE epitranscriptomics. As the present techniques cannot accurately detect TE modifications, focused research is necessary to generate new NGS platforms that can advance the understanding of all types of TE modifications in plants.

Recent molecular biology approaches such as ALE-seq, mobilome-seq, and VLP DNA-seq are more applicable in detecting active TEs in plants [320,321]. However, multi-mapped reads are typically discarded or not considered for analysis because of short-read sequencing. Thus, long-read sequencing technologies have recently been used as promising alternative methods that can easily separate different copies of the same family of TEs. For instance, unique transcripts containing various TEs were identified in maize using PacBio single-molecule RNA sequencing [322]. In addition, Oxford Nanopore Technology (ONT) can generate complete gene-like transcript annotation for TEs [323], suggesting that long-read sequencing allows the mapping of TE reads to a unique position of the plant genome.

However, conventional molecular biology techniques are still commonly used to study TEs. Although some approaches provide unique information, these are not applicable with genome-wide approaches. Although TE-derived transcripts are commonly quantified using qRT-PCR, this method has several major limitations. First, the main portion of the raw material starts with high-quality RNA, which contains pre-mRNA. Accordingly, the process begins with autonomous and passive transcription. Second, it is challenging to develop probes and primers that are truly different for a specific TE family. Third, the order of the amplified fragment cannot be predicted and is more likely to be a shortened transcript [317,324]. Unlike Southern blotting, Northern blotting assesses the size distribution of TE transcripts and whether full-length transcripts are present. Finally, programming individual TE loci with a reporter gene knock-in can be used to measure and parallelize gene expression levels accurately and rapidly [317,325]. This methodology has been employed in measuring each individual *Ty1* RTE present in *S. cerevisiae* [326]; however, the results cannot be easily applied or generalized. Detection of TE proteins is also important. Internal TE mutations often inhibit translation of TE proteins, and post-translational modifications limit RTEs downstream. Western blotting and immunofluorescence experiments can address this issue. However, all conventional molecular biology techniques have several major limitations and advantages [317]. Thus, new approaches are needed to study a genome-wide view of TE expression.

## 17. Recent Machine Learning and Computational Tools for Analysing

Genome-wide analyses of TE methylation are limited due to the complex structures and high diversity of TEs. Several TE-dedicated computational tools (Table 5) are available for genome-wide analysis of TE expression and TE classification. These tools use various approaches, such as structure-based, homology-based, comparative genomics, and *de novo*. However, using these tools can still be challenging due to the polymorphic structures of TEs; thus, there are still debates on TE classification and annotation. No single bioinformatics tool can give reliable results on different types of TEs, and all tools have a high rate of false positives [30,327]. In general, RNA-seq data is mostly used for genome-wide approaches but mapping strategies of TEs with reference genomes mainly differ. Consequently, in addition to computational tools, the use of machine learning algorithms in bioinformatics has rapidly increased in recent years due to their demonstrable achievements in handling the difficult task of managing large datasets. Examples include genome annotation, classification of various plant genotypes with morphological and molecular markers, modularity and prediction of important quantitative properties in plants, analysis of complex, non-linear plant characteristics, and prediction and optimization of in vitro breeding methods. Various types of machine learning have been developed, each with its own methods, strengths, and disadvantages, thus making certain approaches more suited to specific tasks. Machine learning is divided into two categories (supervised and unsupervised), both of which improve the accuracy of TE detection by using results obtained by conventional software [30]. Machine learning can classify autonomous and non-autonomous TEs derived from LTR-RTEs using different features, such as LTR and ORF lengths. This can also distinguish between retroviral LTRs and other RTEs. Using machine learning, it is possible to discover new information on TEs, such as arrays of TEs, new transposition, TE methylation, new ncRNAs, and new DNA motifs. Using machine learning applications, detection of single nucleotide polymorphisms (SNPs) associated with TEs are useful for creating TE population models. Variation in allele frequencies may be used to reveal TE positive selection. However, very few tools, such as Red and TEClass, apply machine learning for TEs and their application in TEs is still limited [30].

Some online TE libraries also use machine learning approaches. For instance, InpactorDB (a semi-curated dataset composed of 130 439 LTR- RTEs from 195 plant genomes of 108 plant species) is an RTE library (e.g., RepeatMasker) for identifying and annotating LTR-RTEs using a machine learning approach [30]. Deep learning is a sub-discipline of machine learning and has shown successful results in genomics; hence, the use of deep learning in machine learning is also rapidly increasing. Deep learning and machine learning are more efficient approaches that use selected histograms or expected histograms to define TE genomic windows and hierarchical classification. However, machine learning has limited potential because of the repetitive nature and diverse polymorphisms of TEs and the species specificity of TEs. Furthermore, although deep learning is useful for genomic research, thus far no software has been developed to use deep learning for the identification and classification of TEs. Despite these challenges, a well-developed machine learning tool for TE classification would advance TE research [327]. Using data mining along with several key features, such as LTR length, TDS, ORFs, TATA boxes, AATAAA, and poly-A tails, developing machine learning for TE classification is possible. Thus, researchers should consider using computational tools and machine learning with deep learning and integrating different TE analyses, which can facilitate development of new applications for TE measurement, transposition, methylation levels, classification, and annotation.

**Table 5:** Analysis of transposable element (TE) unit expression from RNA-seq results using statistical methods and approaches. The table was adapted and recreated from Lanciano et al. [317], with copyright permission from the Licensor Springer Nature (Nature Reviews Genetics: Nature publisher) and Copyright Clearance Center (https://www.copyright.com) (Supplementary File S5).

Approaches or Tools	Mapping or Pseudo-Mapping	Fate of Multimappers	Type of Quantification	Distinguishes Unit-Length Transcripts from other TE-Derived Transcripts	Includes Polymorphic TE Expression	Notes	References
Endogenous retrovirus (ERV) map	Reference genome	Discarded	Locus specific	-	-	Uses a curated full-length human ERV database	[328]
L1EM	Model transcriptome	EM algorithm	Locus specific	+	-	Proof-of-principle on human long interspersed element 1 (L1) could be generalized	[329]
Manual curation	Reference genome	Discarded	Locus specific	+	-	Difficult to generalize	[324]
Multi-omics 1	Reference genome	NA	Locus specific	+	+	Combines targeted DNA sequencing, RNA-seq, and ChIP-seq (chromatin immunoprecipitation followed by sequencing)	[330]
Multi-omics 2	Reference genome	NA	Locus specific	+	+	Combines whole-genome sequencing and RNA-seq	[331]
Random assignment of multimappers	Reference genome	Randomly assigned	Locus specific	-	-	Locus-specific transcription not reliable on youngest TEs	[332]
RE discover TE	Model transcriptome	EM algorithm F	Family specific	+	-	Uses Salmon TE algorithm	[333]
Rep Enrich	Reference genome	Remapped on TE pseudogenome	Family specific	-	-	-	[334]
Salmon TE	Consensus transcriptome	Expectation-maximization (EM) algorithm	Family specific	-	-	Rapid pseudo mapping	[335]
SQuIRE	Reference genome	EM algorithm	Locus specific	-	+/−	Polymorphic insertion can be added as extra chromosome if internal sequence known	[336]
TE tools	TE pseudo genome	Randomly assigned	Family specific	-	-	Applicable to unassembled genomes	[337]
TEcandidates	Reference genome	Remapped on partially masked reference genome	Locus specific	-	-	-	[338]
Telescope	Reference genome	EM algorithm	Locus specific	+	-	-	[339]
TEtranscripts	Reference genome	EM algorithm	Family specific	-	-	Commonly used tool, tested on a wide variety of organisms	[340]
TeXP	Reference genome	Randomly assigned	Family specific	+/-	-	Subtracts signal from pervasive transcription but not from other forms of chimeric transcripts	[341]

## 18. Future Perspectives and Biotechnological Opportunities

Plant research has addressed important questions on whether TE-associated DNA variants contribute to evolutionary transition without affecting the genome. To better understand the impact on evolution, extensive molecular studies on the forms, origins, and impacts of TE activation in *Arabidopsis* have been performed. The results are also applicable to other organisms, especially maize [5]. In particular, the epigenetic and genetic influence of TEs on both hosts and TEs remains relatively understudied. The impact of TEs is attributed to the influence on the genome by suppressing genome recombination in the locality of TEs [226]. In the long term, peripheral transmission effects could theoretically influence overall evolution and have significant implications for genetic and molecular experiments that employ epigenomics [342]. Long-read technologies may elucidate the function of TEs from diverse plants [343]. Similar to DNA methylation, epitranscriptomic modification of RNAs (posttranscriptional RNA modifications) found in eukaryotes is a new layer of gene regulation and may function against TE transcripts [344]. Furthermore, single-cell genomics technologies, for example, appear to be a promising alternative for investigating DNA context in individual cells. Digital droplet PCR (ddPCR) is cost-effective and easy to use [345]. Since ddPCR performs a PCR on many thousands of tiny droplets, the digital presence or absence of TE in each droplet is easily identified by counting the number of droplets. Overall, the latest advances in DNA sequencing have radically changed the direction of transposon research. Relying on new types of epigenomics would open up knowledge and allow engineering of non-genetically modified crops [320].

## 19. Conclusions

It is generally agreed that TEs facilitate genetic and evolutionary diversification. Although some circumstantial evidence supports the above hypothesis, none of it is substantial and there is no direct proof that TEs facilitate ripening inhibitors. TEs are most often thought to create new genetic and phenotypic diversity via the introduction of new regulatory elements and gene and chromosomal disruptions. TEs also often play a crucial role in lineage-specific regulatory and coding sequence evolutions, contributing to new gene functions. Thus, TEs play a key role in the emergence of new phenotypes. For example, TEs are the primary source of novel regulatory sequence variations in primates. Adaptive novelty is mainly due to TE behaviour, which results in a large variety of genetic alterations, such as gene replication, enhanced expression, and newly created genes. Until now, most analyses of TEs only addressed occurrences of TEs and gene activity or transcript and phenotype relationships. A better understanding of the 3D chromatin structure organization within the nucleus may increase our understanding on the function of chromatin structure and its relation to mechanistic genome variations. This review highlighted the need to assess the regulation of TEs and their influence on the adaptive genome. This may facilitate development of improved traits for climate resilience and stress tolerance in the future.

## Supplementary Materials

The following supporting information can be downloaded at: https://www.mdpi.com/1422-0067/22/21/11387/s1. Supplementary File S1 contains the copyright transfer documents of Figure 1 and Figure 4. Supplementary File S2 contains the copyright transfer documents of Table 1. Supplementary File S3 contains the copyright transfer documents of Table 2. Supplementary File S4 contains the copyright transfer documents of Figure 2, Figure 3 and Figure 5. Supplementary File S5 contains the copyright transfer documents of Table 5.
