# Correction: Ramakrishnan et al. The Dynamism of Transposon Methylation for Plant Development and Stress Adaptation. *Int. J. Mol. Sci.* 2021, *22*, 11387

**DOI:** 10.3390/ijms232214107

**Published:** 2022-11-15

**Authors:** Muthusamy Ramakrishnan, Lakkakula Satish, Ruslan Kalendar, Mathiyazhagan Narayanan, Sabariswaran Kandasamy, Anket Sharma, Abolghassem Emamverdian, Qiang Wei, Mingbing Zhou

**Affiliations:** 1 Co-Innovation Center for Sustainable Forestry in Southern China, Nanjing Forestry University, Nanjing 210037, China; 2 Bamboo Research Institute, Nanjing Forestry University, Nanjing 210037, China; 3 Department of Biotechnology Engineering, & The Jacob Blaustein Institutes for Desert Research, Ben-Gurion University of the Negev, Beer Sheva 84105, Israel; 4 Helsinki Institute of Life Science HiLIFE, Biocenter 3, Viikinkaari 1, University of Helsinki, FI-00014 Helsinki, Finland; 5 National Laboratory Astana, Nazarbayev University, Nur-Sultan 010000, Kazakhstan; 6 PG and Research Centre in Biotechnology, MGR College, Adhiyamaan Educational Research Institute, Hosur 635 109, Tamil Nadu, India; 7 Institute for Energy Research, Jiangsu University, Zhenjiang 212013, China; 8 Department of Plant Science and Landscape Architecture, University of Maryland, College Park, MD 20742, USA; 9 State Key Laboratory of Subtropical Silviculture, Zhejiang A&F University, Lin’an, Hangzhou 311300, China; 10 Zhejiang Provincial Collaborative Innovation Center for Bamboo Resources and High-Efficiency Utilization, Zhejiang A&F University, Lin’an, Hangzhou 311300, China

The authors would like to make the following corrections to the original publication [1]. In the original publication, the copyright transfer information for the original illustrations and tables was overlooked. Therefore, the correct legends for the original figures and tables are provided below. The copyright permissions for the figures and tables are also included as Supplemental Files S1–S5. The reference numbers match the reference numbers in the original publication. The authors state that the scientific conclusions are unaffected. This correction has been approved by the Academic Editor, and the original publication has also been updated.

**Figure 1**. Primary regulatory roles of transposable elements (TEs). TEs are a rich source of host genome innovations. TE functions are either harmful or beneficial to the host genome, and their integration in the genome may induce deleterious mutations. Silenced TEs, mostly covered with DNA methylation, can affect the expression of nearby genes. In contrast, active TEs can act as regulatory elements by producing noncoding RNA (ncRNA) and alternative promoters. The illustration was adapted and redrawn from Jönsson et al. [43], with copyright permission from the Licensor Elsevier (Trends in Genetics: Cell Press publisher) and Copyright Clearance Center (https://www.copyright.com) (Supplementary File S1).

**Table 1.** Class- and family-wise examples of transposable elements (TEs) in different plant species. The table was adapted and recreated from Feschotte et al. [64], with copyright permission from the Licensor Springer Nature (Nature Reviews Genetics: Nature publisher) and Copyright Clearance Center (https://www.copyright.com) (Supplementary File S2).

**Table 2.** Proportion of class I and class II transposable elements (TEs) in the total genome of different plant species [99–102,104,110,113–129]. The table was adapted and recreated from Ragupathy et al. [99], with copyright permission from the Licensor Elsevier (Trends in Plant Science: Cell Press publisher) and Copyright Clearance Center (https://www.copyright.com) (Supplementary File S3).

**Figure 2.** Cellular functions of DNA methylation (m) in the plant genome. DNA methylation regulates transposon activation, gene regulation, and chromosome interactions. (**A**) Methylation in the gene promoter either represses or activates transcription [229–233]. (**B**) Gene body methylations mainly occur in the CG context, although its function remains unknown [42,231,234–236]. (**C**) DNA methylation in heterochromatin regions causes the ASI1-AIPP1-EDM2 complex to enhance polyadenylation sites (red stars). ASI1 binds RNA and associates with chromatin, and EDM2 catches demethylated histone H3 lysine in the heterochromatin region [159,237–239]. (**D**) The methylation of transposons and other DNA repeats mainly occurs in pericentromeric heterochromatin regions [231,235]. (**E**) Chromosome interactions among pericentromeric and heterochromatin islands are regulated by DNA methylation, and repressive chromatin regions are characterized by abundant transposons and small RNAs [240,241]. ASI1, anti-silencing 1; AIPP1, immunoprecipitated protein 1; EDM2, enhanced downy mildew 2; POL II, RNA polymerase II. The illustration was adapted and redrawn from Zhang et al. [42], with copyright permission from the Licensor Springer Nature (Nature Reviews Molecular Cell Biology: Nature publisher) and Copyright Clearance Center (https://www.copyright.com) (Supplementary File S4).

**Figure 3.** Functions of transposable element (TE) methylation in plant growth, stomata formation, and fruit ripening. (**A**) In the vegetative cell (male gamete) of *Arabidopsis*, the TE is silenced by DME-mediated DNA methylation by downregulating the chromatin remodeller DDM1. Small interfering RNAs (siRNAs) derived from TE transcripts travel from the vegetative cell to the sperm cells to reinforce global demethylation (m) in the endosperm with reinforced CHH methylation (H represents A, T, or C) [160,242–245]. (**B**) Gene imprinting in the endosperm occurs either at MEGs or PEGs through DNA and histone H3 lysine methylations [246–248]. (**C**) Methylation at the promoter of the gene encoding epidermal patterning factor 2 (EPF2) that suppresses stomata formation is pruned by ROS1, whose mutation silences the EPF2 or the ERECTA genes, thus resulting in stomata formation in *Arabidopsis* [249,250]. (**D**) Gradual expression of DML2 during tomato fruit ripening reduces 5-methylcytosine (mC) DNA methylation at several genes (such as CNR, involved in fruit ripening) and epimutation of those genes inhibits fruit ripening [42,229,251]. DME, transcriptional activator demeter; DDM1, decreased DNA methylation 1; MEGs, maternally expressed genes; PEGs, paternally expressed genes; ROS1, repressor of silencing 1; DML2, DNA demethylase DME-LIKE 2; MET1, methyltransferase 1. The illustration was adapted and redrawn from Zhang et al. [42], with copyright permission from the Licensor Springer Nature (Nature Reviews Molecular Cell Biology: Nature publisher) and Copyright Clearance Center (https://www.copyright.com) (Supplementary File S4).

**Figure 4.** Transposable elements (TEs) are suppressed by DNA and histone methylations. (**A**) TE methylation is most commonly found in the CG context. The de novo DNA methylation is performed by DNA methyltransferases DNMT3A and 3B; the pattern of DNA methylation is maintained by DNMT1 by adding a methyl group to the newly synthesized DNA strand (a complementary strand of the hemi-methylated DNA strand), thus ensuring that the epigenetic modifications are inherited by the daughter cell. (**B**) Nucleosomes are made up of DNA and eight histone proteins. These proteins can be modified in several ways for chromatin accessibility, thereby either activating or inactivating gene expression (gene imprinting). TRIM28, a silencing complex, recognizes KRAB-ZNFs (Kruppel-associated box zinc-finger proteins), which contain a TE-binding domain and deposits H3K9me3 on TE (euchromatin region), thus causing TE repression and heterochromatin formation. The illustration was adapted and redrawn from Jönsson et al. [43], with copyright permission from the Licensor Elsevier (Trends in Genetics: Cell Press publisher) and Copyright Clearance Center (https://www.copyright.com) (Supplementary File S1).

**Figure 5.** Epigenetic modifications under stress conditions and possible stress memory. (**A**) Both biotic and abiotic stresses can induce or change DNA methylation (5-methylcytosine, mC) and induce other epigenetic changes in the genome; such modifications are associated with the expression of stress-response genes, which conversely may lead to epigenetic processes. Reprogrammed epigenetic modifications (stress memory) are inherited by the offspring. (**B**) In *Arabidopsis*, ROS1, DML2, and DML3 remove DNA methylation, thus collectively regulating stress responsive genes in their vicinity. Defects in demethylases, such as ROS1, DML2 and DML3, exhibit increased susceptibility to the fungal pathogen *Fusarium oxysporum* [315]. (**C**) During *Arabidopsis* recovery from heat stress, DDM1 and MOM1 regulate the deletion of stress-induced epigenetic memory. Mutations in DDM1, a chromatin remodeller, assuages transcriptional silence with a significant loss of DNA methylation. MOM1 intermediates facilitate transcriptional silence via an unknown mechanism without loss of DNA methylation. Dysfunction of DDM1 and MOM1 in heat stress-induced gene de-silencing can be inherited in plants exposed to repeated stress [316]. ROS1, repressor of silencing 1; DMEL2 and DML3, transcriptional activator demeter (DME)-Like 2 and 3, respectively; DDM1, decreased DNA methylation 1; MOM1, morpheus molecule 1; H3K9me2, demethylated histone H3 lysine 9. The illustration was adapted and redrawn from Zhang et al. [42], with copyright permission from the Licensor Springer Nature (Nature Reviews Molecular Cell Biology: Nature publisher) and Copyright Clearance Center (https://www.copyright.com) (Supplementary File S4).

**Table 5.** Analysis of transposable element (TE) unit expression from RNA-seq results using statistical methods and approaches. The table was adapted and recreated from Lanciano et al. [317], with copyright permission from the Licensor Springer Nature (Nature Reviews Genetics: Nature publisher) and Copyright Clearance Center (https://www.copyright.com) (Supplementary File S5).

**Supplementary Materials:** The following supporting information can be downloaded at: https://www.mdpi.com/article/10.3390/ijms222111387. Supplementary File S1 contains the copyright transfer documents of Figures 1 and 4. Supplementary File S2 contains the copyright transfer documents of Table 1. Supplementary File S3 contains the copyright transfer documents of Table 2. Supplementary File S4 contains the copyright transfer documents of Figures 2, 3 and 5. Supplementary File S5 contains the copyright transfer documents of Table 5.
